# Lung Microbiota: Its Relationship to Respiratory System Diseases and Approaches for Lung-Targeted Probiotic Bacteria Delivery

**DOI:** 10.1021/acs.molpharmaceut.3c00323

**Published:** 2023-06-21

**Authors:** Nilufer Yuksel, Busra Gelmez, Ayca Yildiz-Pekoz

**Affiliations:** † Department of Pharmaceutical Technology, Faculty of Pharmacy, Ankara University, 06560 Ankara, Turkey; ‡ Department of Pharmaceutical Technology, Faculty of Pharmacy, Istanbul University- Cerrahpasa, 34500 Istanbul, Turkey; § Graduate School of Health Sciences, Ankara University, 06110 Ankara, Turkey; ∥ Department of Pharmaceutical Technology, Faculty of Pharmacy, Istanbul University, 34116 Istanbul, Turkey

**Keywords:** probiotics, *Lactobacillus*, respiratory tract, lung microbiota, chronic lung diseases, probiotic technology, microencapsulation

## Abstract

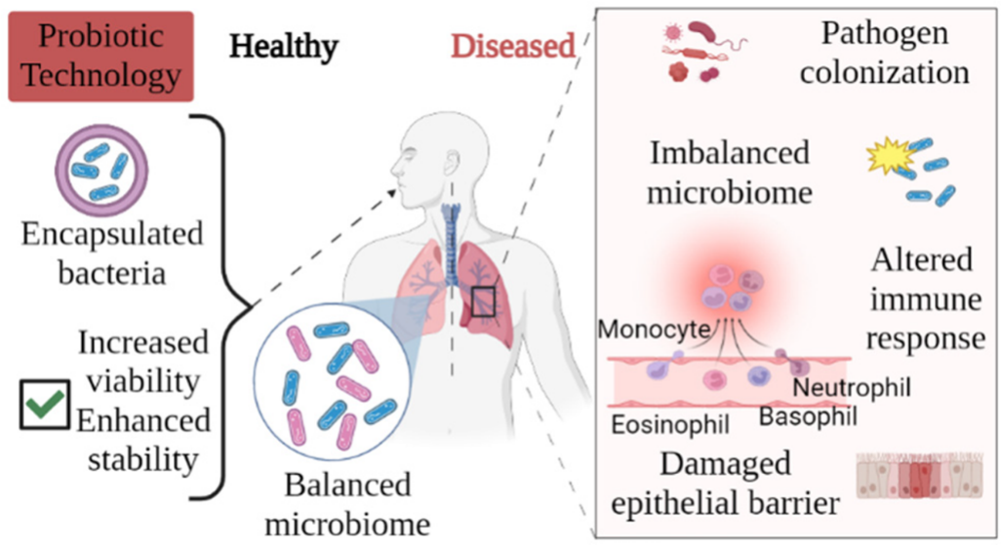

Microorganisms that make up the local microbiota (such as *Lactobacillus* sp. and *Bifidobacterium* sp.) play a crucial role in the modulation of diseases and health states by taking place not only in the gut but also in many parts of our body. There is also interference between the gut and the lung via the gut–lung axis. The relationship between respiratory diseases and lung microbiota, which become more of an issue of particular importance in recent years, shows that probiotics play an essential role in maintaining the balance of microorganisms in the respiratory tract. However, studies on probiotics’ prophylactic or therapeutic application in chronic lung diseases are limited. In this review, the literature between 1977 and 2022 was surveyed. General information about human microbiota was accessed in earlier sources, and especially in the past decade, research on lung microbiota has been reached. The relationship between lung microbiota and important respiratory diseases such as bronchopulmonary dysplasia, chronic obstructive pulmonary disease, pneumonia, cystic fibrosis, allergy-asthma, influenza, lung cancer, and COVID-19 infection, was scrutinized after mentioning human microbiota, the gut–lung axis, and respiratory tract microbiota. The mechanism of action of probiotics and the formulation approaches of probiotics in terms of pharmaceutical technology were reviewed. Finally, future perspectives on lung-targeted administration of probiotic bacteria with prophylactic or therapeutic potential, or both, were presented.

## 1 Introduction

The human microbiota is a living system that contains trillions of microbial cells and genes. While the microbiota is defined as a “microbial taxon associated with humans”, the microbiome is a “community containing their microorganisms and genes”.^1,2^ The human microbiota is a dynamic ecosystem that has leading roles in various physiological, psychological, metabolic, and immunological processes and begins to take shape from birth.^3^

This adventure, which started with the “*Human Microbiome Project*”, is further illuminated by studies carried out every day. In the frame of this project, which began in 2008, samples were taken from different parts of the body. Then a taxonomic profile was created with 16S rRNA gene sequences.^4^ The main goal of this project (led by the US National Institutes of Health) was to examine whether human microevolution affects their health and, therefore, their susceptibility to diseases. Besides, it aimed to contribute to the development of the food and pharmaceutical industry by examining the microorganisms in the human microbiota and the chemicals produced by these microorganisms.^5^

**Figure 1:**
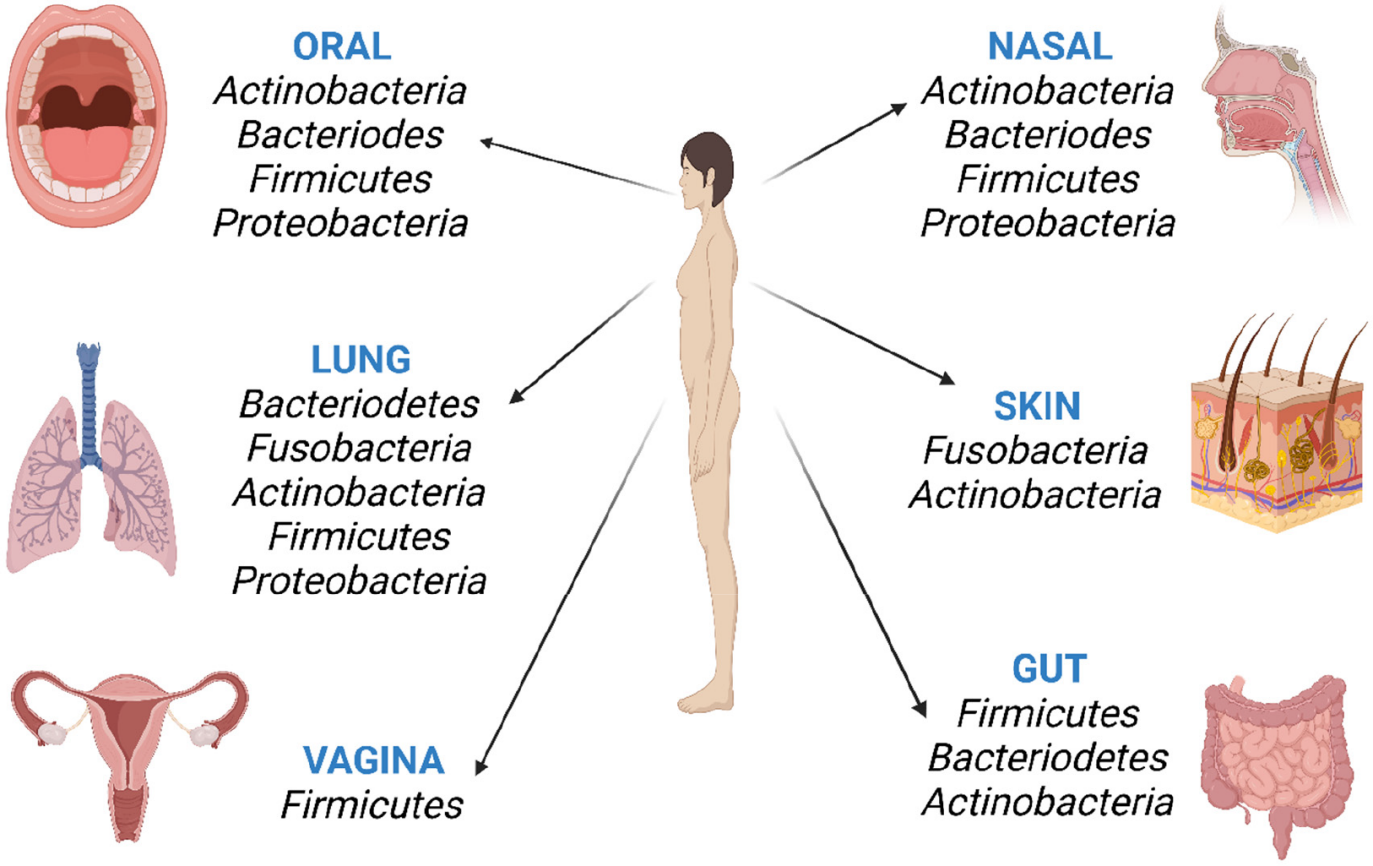
Major phyla originating from the microbiota of different regions in the human body.

Trillions of symbiotic microorganisms live in many human body regions, especially in the gut, skin, mouth, stomach, vagina, lung, and respiratory tract.^6^ Bacterial phyla located in different body parts are listed in Figure 1. Their families and species found in significant proportions in the body are presented in Table 1. The symbiotic relationship between the gut microbiota and the host is regulated and maintained by a complex network of interactions involving metabolic, immune, and neuroendocrine crosstalk.^4,7^ A healthy microbiota and the immune system play a leading role in body homeostasis. Conversely, dysbiosis occurs when homeostasis is disrupted due to an imbalance in the microflora. This disturbed microbiota balance causes the proliferation of pathogens, impaired immune response, and tissue damage. In the case of regeneration and balancing of the microflora, the immune response increases, and tissue damage is recovered.^8^

**Table 1:** Examples of Phylum, Families, and Species Found in Significant Proportions in Humans

phylum	family	genus	species	refs
*Firmicutes*	*Lactobacillaceae*	*Lactobacillus*	*L. acidophilus*	(9)
			*L. rhamnosus*	
			*L. casei*	
			*L. paracasei*	
			*L. plantarum*	
			*L. salivarius*	
	*Bacillaceae*	*Bacillus*	*B. acidiprudens*	(10)
			*B. infantis*	
	*Clostridiaceae*	*Clostridium*	*C. difficile*	(10)
			*C. vulturis*	
	*Lachnospiricaea*	*Eubacterium*	*E. siraeum*	(11)
			*E. rectale*	
	*Ruminococcaceae*	*Ruminococcus*	*R. flavefaciens*	(11)
			*R. bicirculans*	
			*R. bromii*	
			*R. champanellensis*	
	*Staphylococcaceae*	*Staphylococcus*	*S. epidermidis*	(12)
			*S. capidis*	(13)
			*S. hominis*	(14)
			*S. aureus*	
*Bacteriodetes*	*Prevotellacea*	*Prevotella*	*P. brevis*	(11)
			*P. ruminicola*	(15)
			*P. bryantii*	
			*P. copri*	
*Actinobacteria*	*Bifidobacteriaceae*	*Bifidobacterium*	*B. breve*	(11)
			*B. adolescentis*	(16)
			*B. longum*	
			*B. bifidum*	
	*Propionibacteriaceae*	*Propionibacterium*	*Pr. acnes*	(17)
	*Corynebacteriaceae*	*Corynebacterium*	*C. simulans*	(13)
			*C. resistans*	(14)
			*C. tuberculostearicum*	
*Fusobacteria*	*Fusobacteriaceae*	*Fusobacterium*	*F. alocis*	(18)
			*F. periodontium*	(19)
			*F. simiae*	(20)
			*F. mortiferum*	
			*F. sulci*	
			*F. nucleatum*	
			*F. necrophorum*	
*Proteobacteria*	*Enterobacteriaceae*	*Eschericia*	*E. coli*	(21)
		*Citrobacter*	*C. europaeus*	(22)
			*C. brakii*	(23)
			*C. youngae*	
			*C.freundeii*	

Probiotics are widely used to restore the microbiota balance and prohibit dysbiosis. Microbial species commonly used as probiotics include the genera *Lactobacillus*, *Streptococcus*, and *Bifidobacterium*, listed as GRAS. Among them, *Lactobacillus* produces chemicals that inhibit pathogenic bacterial growth and/or infectivity, e.g., organic acids such as lactic acid, bacteriocins, and hydrogen peroxides. It has been observed that lactic acid bacteria have a prophylactic effect against some diseases and strengthen the human immune system.^24−26^*Bifidobacterium* species are the crucial bacteria that make up human gut microbiota. *Bifidobacterium* has been shown to have many beneficial effects on humans and animals, such as antibacterial, anti-inflammatory, and antidepressant effects, host immune system regulation, and host nutrition (such as vitamins and calcium ions) adsorption. At the same time, these species can produce bioactive compounds such as vitamins, essential fatty acids, and exopolysaccharides.^16,27^

Probiotics are mainly used in gastrointestinal (GI) and inflammatory diseases such as antibiotic-related diarrhea and inflammatory bowel disease (IBD). Besides, there are many studies on the effectiveness of probiotics in acute and chronic respiratory diseases. It is emphasized that maintaining the gut microbiota balance in the oral administration of probiotics is also effective in lung diseases through the gut–lung axis. The use of probiotics in respiratory tract diseases is relatively new. In this review, we will focus on lung microbiota and their relationship with diseases, the utilization of probiotics in the respiratory system, and the relationship between probiotics and microbiota will be mentioned. Afterward, pharmaceutical technological applications will be presented to deliver probiotics to the body and potential approaches for targeted probiotic administration to the lungs.

## 2 Lung Microbiota and Relationship with Diseases

### 2.1 Gut–Lung Axis

The gut is an organ described as the second brain, which enables the regulation of diseases and health conditions and the presence of microorganisms in our body through the axis including both the brain and the lung.^8,28^ As a result of the communication of the gut with other organs through biochemical signals, many diseases are associated with deteriorated gut microbiota. In addition, the dietary habits and phylogeny of the host further contribute to the microbial community of the gut in humans and other mammals.^4^

**Figure 2:**
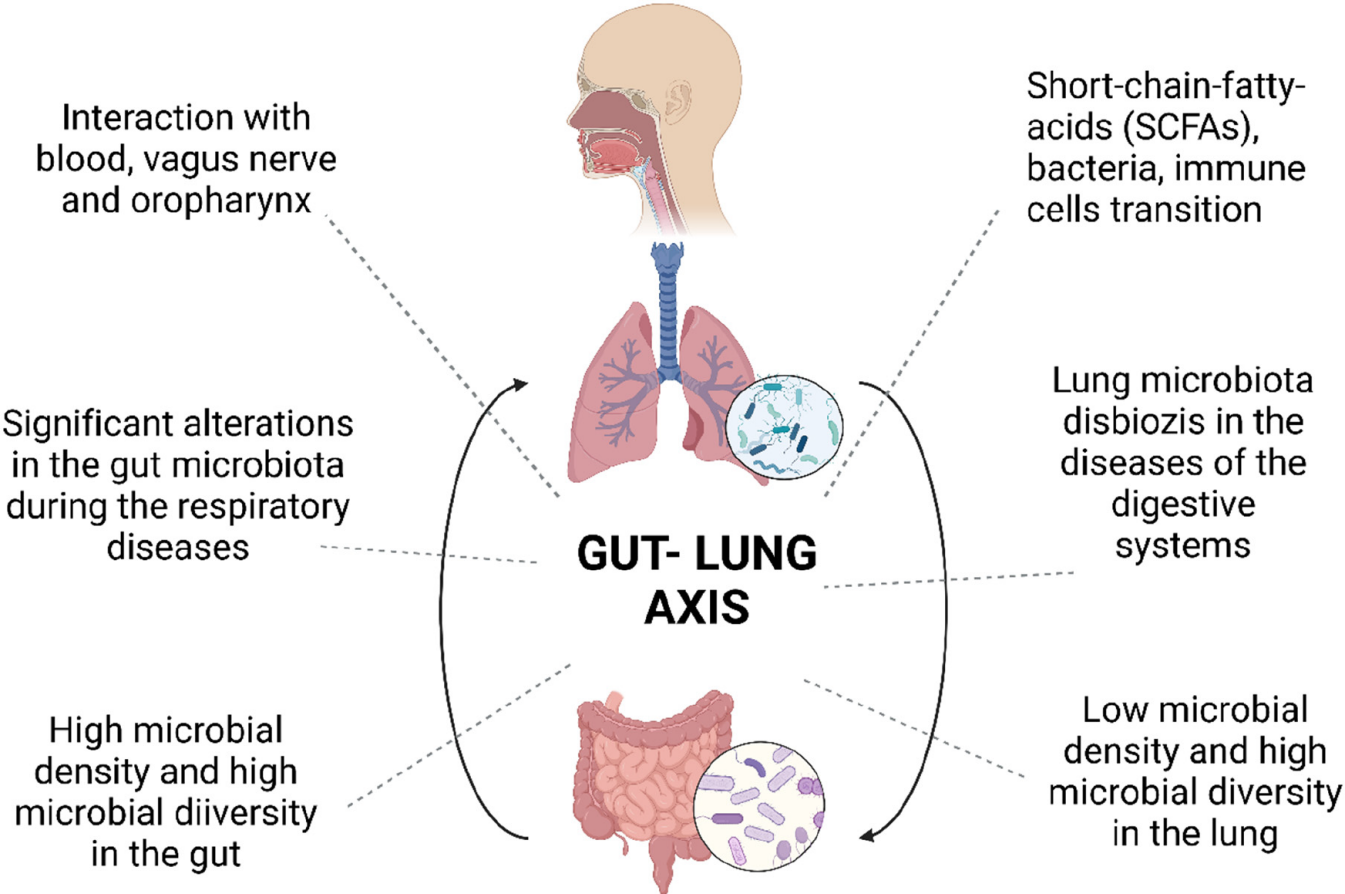
Schematic diagram of the bidirectional crosstalk between gut microbiota and the lungs.

The lung and gut are two organs that are similar at the level of the epithelial structure. However, they vary regarding microorganism density and microbiota diversity.^8,29^ Two-way communication (crosstalk) between the gut and lung is provided via the oropharynx, blood, and vagus nerve (Figure 2). Environmental products and bacteria can pass from one organ to another through the oropharynx. Bacterial particulates, short-chain fatty acids (SCFAs), and immune cells are transmitted through the blood. The vagus nerve realizes interaction through mutual signals. Thus, gut and lung ecosystems are significantly linked to nutrition, respiratory, and digestive health and immune defense through a complex system of intercommunication.^30^

Dietary fermentable fiber content has been found to alter the gut and lung microbiota composition, mainly by affecting the ratio of *Firmicutes* to *Bacteroidetes.* The level of circulating SCFAs increases due to fiber metabolism in the gut microbiota. SCFAs enhance the formation of macrophages, dendritic cell precursors, and, subsequently, seeding of dendritic cells in the lung. Ultimately, it can affect the severity of allergic inflammation and shape the immunological environment in the lung.^31,32^

Schuijt et al. investigated the function of the host gut microbiota against *Streptococcus pneumonia* infections in gut microbiota-depleted mice. While bacterial spread, inflammation, organ failure, and increased mortality were observed in these mice with pneumonia, it was shown that fecal microbiota transplantation to these mice boosted primary alveolar macrophage function against pneumococcal pneumonia and played a role as a protective factor in host defense.^33^ In addition, acute changes in the intestinal microbiota were observed in mice in which lipopolysaccharides were administered by intratracheal instillation to induce lung damage by stimulating the inflammatory response.^34^ The results of the studies prove that the gut–lung axis of bacteria is bidirectional.

### 2.2 Lung Microbiota

From infancy, the mode of delivery (normal birth/cesarean section), breast milk, genetic factors, vaccines, antibiotic use, and the environment play a meaningful role in shaping the respiratory tract and lung microbiota.^35^ Lungs were not examined in the first microbiome studies and were considered sterile. However, studies performed in recent years have shown that the lungs are not sterile and have hosted many microorganisms since birth.^29,36^ Innate and adaptive host defenses, such as inhalation, mucociliary clearance, and coughing, impact the lung microbiota. Oxygen, pH, blood perfusion, and the number of inflammatory cells are other factors that contribute to the determination of the local microbiome.^37^ Humans breathe bacterial cells at a rate of 10^4^–10^6^ units/m^3^ during the day. In addition, the upper respiratory system (URT, i.e., the nasal cavity, sinuses, nasopharynx, and oropharynx) is exposed to atmospheric physical and chemical parameters, including changing humidity, oxygen, temperature, immunological factors, and nutrients. Along with anatomy, these factors shape specific microenvironments in the URT to harbor different microbial communities consisting of permanent and transient microorganisms in varying proportions.^38^ Whereas microbial replication is in balance in healthy lungs, this balance is disrupted in respiratory diseases. Lung microbiota are defined as a dynamic microbiota according to “The Yin-Yang Phenomenon”. According to this phenomenon, the lungs have a transient but not resident microbiome, and there is a dynamic balance between the transient microbiome of the lower respiratory system (LRT) and immune responses.^36,39^

Sampling from the lungs is performed by bronchoscopy and the bronchoalveolar lavage (BAL) method. In the BAL process, saline from the bronchoscope’s tip is administered to the lungs, and the liquid is aspirated back. While classical culture methods were applied to this liquid before, innovative approaches are now used, and the microflora composition is assayed by DNA extraction, 16S rRNA quantitation, and sequence analysis. Prokaryotic ribosomes have the 30S and 50S subunits, and the 16S rRNA gene is located in the 30S subunit. In addition to regions shared among bacterial species, this gene contains nine regions (V1–V9) with high variability between taxa. 16S rRNA sequence analysis targets these regions. Thus, it provides phylogenetic information for comparing microbial diversity in environmental samples.^30,39−41^ Charlson et al. (2011) stated that molecular-based studies standing only on BAL collection or the use of mucosal brushes for identifying lung microbiota resulted in a characteristic microbial distribution at very high levels in the airways of healthy individuals. However, they emphasized that a separate analysis of the URT microbiota and environmental additive controls is required to determine the microbial population of LRT.^42^ Therefore, researchers analyzed bacterial mass and composition by 16S rDNA Q-PCR (quantitative polymerase chain reaction) and deep sequencing on six healthy individuals’ samples taken from different parts of the respiratory tract. URT was sampled with oral washing and oro-/nasopharyngeal swabs. Two bronchoscopes were used for sample collection up to the glottis, followed by bronchoalveolar lavage and mucosal sampling with a protected specimen brush. The study results showed that the lung microbiota has decreasing biomass from URT to LRT, that the lung community of each individual is very closely related to their URT microbiota, and that the healthy LRT bacterial population has primarily reflected URT microorganisms. Generally, there are species in the *Firmicutes* (including *Streptococcus* sp. and *Veillonella* sp.), *Bacteriodetes* (including *Prevotella* sp.), and *Proteobacteria* phyla in the adult lung microbiota.^35,37,43^

### 2.3 The Relationship of Respiratory Diseases with Lung Microbiota and Probiotic Use

Over the years, the incidence of autoimmune lung diseases has increased due to dietary habits, lifestyle changes, antibiotic use, and decreased exposure to the external environment. This situation might contribute to the decrease in immunity.^43^ As explained in the “hygiene theory”, people less exposed to microorganism diversity, especially during infancy and childhood, have a higher risk of developing allergic and autoimmune diseases in the future. It has been proven that babies born to a farming and animal husbandry family have a lower risk of developing asthma and allergies in the future.^30^ Okada et al. have defined the mechanisms of hygiene theory as the disruption of the balance between T helper type-1 (Th1) and T helper type-2 (Th2) cells, the reduction of immunological response to infectious agents through stimulation of antigenic competition and nonantigen-specific receptors, and the relationship between the polymorphism of various genes encoding molecules in immune responses, allergies, and autoimmune diseases.^44^

**Figure 3:**
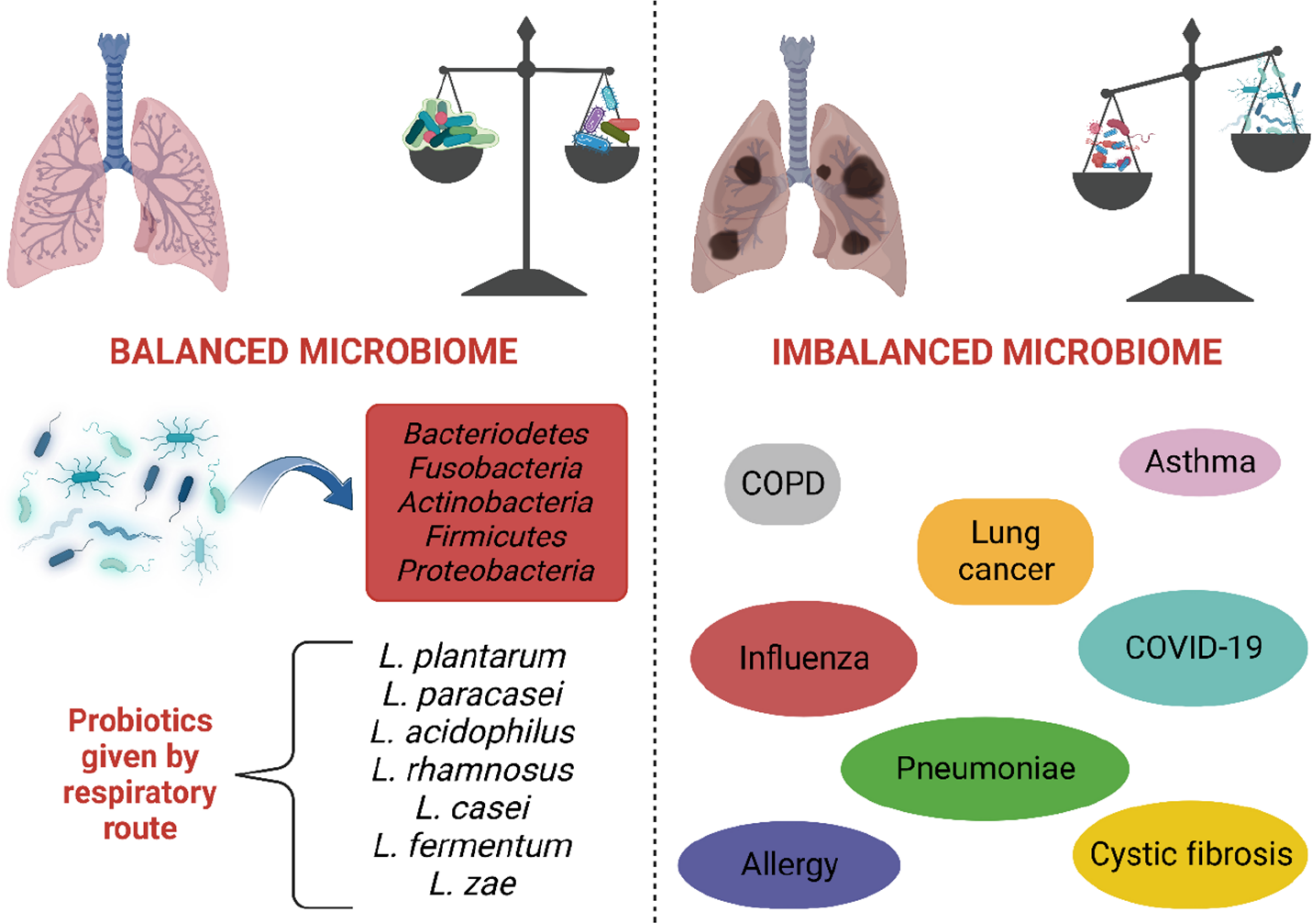
Healthy lung, lung microbiota, and its relationship with diseases. The balance of microorganisms in the lungs is related to health. Interruption of microorganism balance and impaired microbiota balance can be the cause or result of lung diseases. In different *in vivo* studies, examples of probiotics given to the respiratory tract by oral inhalation or the intranasal route are shown in the figure.

Antibiotics in treating various bacterial diseases reduce bacterial diversity and the number in the respiratory tract.^45^ This situation may change the response to drugs and clinical features. In addition, many factors, such as anatomical injuries, pathological effects, physiological changes, and immune system defects, can cause lung dysbiosis and chronic lung diseases. In cases where the balance of microorganisms is disturbed and dysbiosis is observed, a basis for various respiratory diseases is formed (Figure 3). However, it is unclear whether dysbiosis is the onset or progression of the disease or the cause/consequence of the immune disorder.^2^

Chronic obstructive pulmonary disease (COPD), asthma, cystic fibrosis (CF), idiopathic pulmonary fibrosis, and allergic rhinitis are among the most commonly observed and complex respiratory diseases with no specific treatments until now.^2,41,46^ Many studies show the effect of dysbiosis in these diseases. The beneficial effects of probiotics are primarily studied in the GI tract. However, recent findings point to these bacteria’s potential to prevent and treat chronic airway diseases. The studies on microbiota change and probiotic application during these diseases are mentioned below.

#### 2.3.1 Bronchopulmonary Dysplasia

Bronchopulmonary dysplasia (BPD) is a chronic lung disease that affects premature babies receiving respiratory support with mechanical ventilation. It is a respiratory failure that occurs shortly after birth and gradually increases in severity in the first 2 days of life.^47^ Lal et al. investigated the airway microbiome in tracheal aspirate samples of extremely preterm and term infants and preterm infants with BPD immediately after birth. Neonates born by cesarean or vaginal delivery have been found to have similar airway microbiomes. However, the diversity of the airway microbiota in infants with BPD was low and very different from those of the other two groups of infants. *Firmicutes* and *Fusobacteria* decreased, while *Proteobacteria* increased in infants with BPD. The level of *N*-acetyl-proline-glycine-proline (Ac-PGP), which causes inflammation, was found to be higher. According to cytokine analysis results performed after inoculating cells with different *Lactobacillus* species, the most substantial anti-inflammatory effect was observed with the combination of *L. plantarum*, *L. acidophilus*, and *L. rhamnosus*, demonstrating its potential use. Moreover, it was observed that neutrophilic inflammation decreased and lung functions improved as a result of inhalation of the *Lactobacillus* combination in mice with BPD, COPD, and CF.^48^

#### 2.3.2 Chronic Obstructive Pulmonary Disease (COPD)

The prevalence of COPD has been increasing in recent years and is the third leading cause of death worldwide with an economic burden. It was found that *Actinomycetes* spp. were dominant in mild/moderate COPD, as *Haemophilus influenza* was dominant in highly severe COPD. Besides, *Proteobacteria* species are prevalent in phlegm content, while the ratio of *Actinobacteria*, *Clostridia*, and *Bacteroides* has decreased.^46,49^ These levels have changed during the use of antibiotics or corticosteroids.^50^ Persons with phlegm samples high in *Haemophilus* and *Streptococcus* have been associated with higher mortality.^39^

The effect of *L. rhamnosus* on COPD-induced mice inhaling cigarette smoke was investigated. *L. rhamnosus* was administered daily to mice 1 week before COPD induction, then three times a week by gavage until euthanasia. After euthanasia, the BAL fluid and the lungs were removed, and then inflammatory parameters were evaluated. In the *L. rhamnosus* group, the passage of inflammatory cells into the airways was inhibited. While exposure to cigarette smoke caused an increase in the level of pro-inflammatory cytokines such as IL-1β, IL-6, TNF-α, IL-17, and TGF-β in BAL fluid, there was a significant decrease in the level of cytokines in the *L. rhamnosus* group. When lung tissues were compared, peribronchial inflammation, alveolar growth, collagen deposition, and destruction of elastic fibers were decreased in the *L. rhamnosus* group; no significant difference was observed between the *L. rhamnosus* group and the control group in the morphometric studies. Exposure of murine and human epithelial cells to cigarette smoke extract resulted in pro-inflammatory cytokines and chemokines secretion. Probiotic stimulation made refractory epithelial cells resistant to inflammatory provocation by cigarette smoke extract, indicating that it can enhance the lung inflammatory response in COPD.^51^

#### 2.3.3 Pneumonia

The bacteria *Streptococcus pneumoniae* and *H. influenza* type B and respiratory virus (RSV) are the most commonly known causes of pneumonia. The spread of antibiotic resistance is a significant public health problem and requires alternative treatments to antibiotics. Clinical studies demonstrate the ability of *Lactobacilli* to prevent pneumonia when administered orally, possibly via the gut–lung axis. The direct administration of probiotics to the respiratory system is being studied in a murine model as a new application.

In a study, first, the probiotics were administered to mice by intratracheal instillation as a prophylactic, and then an infection with *Pseudomonas aeuruginosa* administered by the same route was induced. The anti-infective activity of the mixture containing equal proportions of probiotics, *L. fermentum*, *L. paraesei*, and *L. zeae*, which is effective against *P. aeuroginosa* in the laboratory environment, was evaluated. Administration of the probiotic mixture to the mice reduced the logarithmic increase rate of *P. aeruginosa* and provided inhibition of virulence factors. In addition, inflammatory cytokines decreased, and cell viability increased.^52^

Zelaya et al. emphasized the association of acute respiratory system infections with increasing acute ischemic heart disease, stroke, and venous thromboembolism. The researchers investigated the effect of probiotic *L. rhamnosus* in nasal administration on the immunocoagulative response during pneumococcal infection in immunocompetent mice and the mechanism of the immunomodulatory effect. Mice infected with *S. pneumoniae* without probiotic treatment had increased concentrations of LDH (lactate dehydrogenase) and albumin in BAL used to assess lung injury. The nasal administration of *L. rhamnosus* significantly reduced the values of LDH and albumin. Nasal probiotic administration beneficially modulated the immune response. It increased the local production of TNF-α and IFN-γ and reduced the tissue damage induced by pneumococcal infection. It also raised the level of IL-10 in both the lungs and the blood. The augmentation in IL-10 level contributes to regulating the procoagulant and antifibrinolytic effects of pro-inflammatory mediators induced against infection.^53^

RSV is an important pathogen causing bronchitis and pneumonia, especially in infants and older adults. RSV might infect different types of cells, mainly epithelial cells, in the respiratory tract. Unfortunately, no approved RSV vaccine is available; the only FDA-approved treatment is prophylactic treatment using a monoclonal antibody, palivizumab.^54^ A study investigated the efficacy of probiotic administration against pneumovirus infection in mice infected with pneumovirus. Researchers have found that protection by *Lactobacillus* can be sustained for up to 5 months with a 40% survival rate and that at least two intranasal inoculations with *L. plantarum* or *L. reuteri* are required for protection against lethal viruses.^55^

#### 2.3.4 Cystic Fibrosis (CF)

CF is an inherited respiratory disease with bronchiectasis and obstructive features that significantly affects the lungs.^56^ In this disease, secretions become thickened, the diversity of microorganisms in the lungs varies, and infection occurs due to progressive pathogenic microorganism airway colonization. The lung microbiota of individuals with CF disease are characterized by a marked increase in the phylum *Proteobacteria*, which includes the typical CF pathogens *Pseudomonas*, *Burkholderia*, and *Haemophilus*, with a further rise in the *Actinobacteria* phylum. In addition, gut microbiota shifts have also been reported in CF patients.^6,57^

Oral *Lactobacillus* supplementation in CF has been reported to reduce intestinal inflammation and prevent pulmonary deterioration by acting outside of the GI tract. *Lactobacillus* isolates have been shown to inhibit *P. aeruginosain vitro* and to improve lung alveolar structure by inducing more and smaller alveoli in germ-free mice. A study of the phlegm of CF patients examined the *Lactobacillus* population. The commonly observed species were *L. rhamnosus*, *L. fermentum*, *L. paracasei*, and *L. gasseri*.^58^ The same researchers screened the *Lactobacillus* strains isolated from their studies *in vitro* in terms of their ability to reduce the synthesis of *P. aeruginosa*-dependent virulence factors (pyocyanin and elastase). They prepared the three *Lactobacillus* strains (*L. rhamnosus* and two *L. fermentum* strains) that they found to be the most effective and the three *Lactobacillus* strains (*L. paracasei*, *L. salivarius*, and *L. brevis*) that they found ineffective as two separate mixtures, including equal proportions of bacteria. Both bacterial suspensions were administered intranasally 18 h before infection with *P. aeruginosa*. Interestingly, both increased the survival rate of mice from 12% to 71% (effective *in vitro*) and 100% (ineffective *in vitro*), independent of *in vitro* anti-*Pseudomonas aeruginosa* activity. These results proved that intranasal preadministration of lactobacilli plays a prophylactic role and prevents fatal complications caused by *P. aeruginosa*.^59^

Coffey et al. reviewed clinical studies conducted to determine the efficacy and safety of probiotics for improving health outcomes in children and adults with CF. In clinical studies of CF, it has been observed that *Lactobacillus* spp., *Bifidobacterium* spp., *Saccharomyces* spp., and *Streptococcus* spp. are generally administered orally as single- or multistrain formulations. The authors concluded that probiotics significantly reduce fecal calprotectin (a marker of intestinal inflammation) in children and adults, may make little or no change in pulmonary exacerbation rates, and are related to some adverse effects including vomiting, diarrhea, and allergic reactions. The authors additionally noted that patients and healthcare providers might consider probiotics; however, further studies and validation are required for the results obtained.^60^

#### 2.3.5 Allergy and Asthma

In many studies of asthma patients, it was observed that the composition of the lung microbiota was different from the healthy group. The *Chlamydia pneumoniae* rate was high in phlegm samples of asthma patients. Colonization of *S. pneumoniae* and *H. influenza* in the URT was found to be associated with increased eosinophilia and IgE concentration.^46^ Less exposure to *Firmicutes* and *Bacteroidetes* may predispose them to asthma and wheezing.^37^ Asymptomatic newborns with colonized *Haemophilus*, *Moraxella*, and *Neisseria* species in their throats have an increased risk of asthma and recurrent wheezing in the first few years of life. The presence of these microorganisms in the respiratory tract is also associated with the exacerbation of asthma and COPD. Hilty et al. found that the pathogenic microorganism *Proteobacteria* (especially *Haemophilus* spp.) was present at a higher rate in the bronchi of adult patients with asthma or COPD and asthmatic children compared to the control group.^61^

The airway microbiota of 65 patients with suboptimally controlled asthma using inhaled corticosteroids were compared with the airway microbiota of 10 healthy individuals by 16S rRNA microarray and parallel clone library sequence analysis. Compared to the control group, the airway bacterial load and microbiota diversity increased in asthmatic patients and were associated with increased bronchial hypersensitivity. In addition, the relative abundance of particular phylotypes, including *Comamonadaceae*, *Sphingomonadaceae*, *Oxalobacteraceae*, and other bacterial families, has been observed to be highly correlated with the degree of bronchial hypersensitivity.^62^

Using intranasal probiotics for treating and preventing allergic sensitization and disease has been identified as a promising strategy. The effects of intranasal administration of two strains of probiotic *L. rhamnosus* (*GG* and *GR-1*) as a preventive treatment in a mouse model of allergic airway disease were investigated. A probiotic suspension at the concentration of 5 × 10^8^ colony forming units (CFUs) in 10 μL of phosphate-buffered saline (PBS) was administered as intranasal drops eight times a day on days 1–4 and 8–11. Afterward, their efficacy was investigated in mice with birch pollen-induced asthma. *L. rhamnosus GG* strain significantly reduced the levels of eosinophils in BAL fluid and the levels of Th2-related cytokines IL-5 and IL-13 in lung homogenates. In addition, airway hyperreactivity was significantly diminished. Furthermore, significantly more viable *L. rhamnosus GG* cells were retained in the nasal mucosa, indicating protection, colonization, and translocation of the instilled probiotic from nasal mucociliary clearance. This finding also explains why *L. rhamnosus GG* strain prevents allergic asthma in a strain-specific manner in a mouse model.^63^

#### 2.3.6 Influenza

Influenza is a viral disease whose management is prioritized by public health authorities worldwide. Although vaccination is applied in practice as a preventive measure, the effectiveness of these vaccines can not be achieved in the next year due to rapid viral mutagenesis. Therefore, it is stated that improving natural defenses by activating cellular immunity may be an effective way to control influenza. Prior intranasal administration of the probiotic *L. rhamnosus GG* to mice reduced symptoms and increased survival percentages in mice infected with the influenza virus (PR8, H1N1). Studies have shown that certain strains of *Lactobacilli*, such as *L. casei* Shirota, characteristically stimulate lung natural killer (NK) cells. The cytotoxic activity of lung cells isolated from mice treated with *L. rhamnosus GG* increased compared to those treated with PBS alone. This result was based on the activation of lung NK cells. In addition, IL-1β, TNF, and MCP-1 mRNA levels included in cellular immunity were significantly higher in lung cells isolated from probiotic-treated mice than in those from control.^64^ The study’s findings demonstrate the potential protective efficacy of *L. rhamnosus GG* by intranasal administration against influenza. In another study, the antiviral protective effects of pretreatment with heat-killed *L. casei* were investigated in influenza-virus-infected mice. Intranasal administration of *L. casei* provided strain-nonspecific protection against different subtypes of influenza virus (H3N2 virus and H1N1 pandemic virus) with a reduction in weight loss, viral load, and survival of all mice. Alveolar macrophage cells increased, levels of proinflammatory cytokines decreased, and early stimulation of virus-specific antibodies occurred.^65^ In another study conducted by Hori et al., after intranasal administration of heat-killed *L. casei Shirota* to mice, the virus titer in nasal wash fluid in mice infected with influenza virus (PR8 and H1N1) was found to be significantly lower than in mice that were not administered probiotics. After completion of intranasal probiotic administration, mediastinal lymph nodes, which play a role in preventing the influenza virus, were removed, and node cells were cultured with and without PR8. Interestingly, cytokine production such as IL-12, IFN-γ, and TNF-α was induced in these cells as was *in vivo* virus infection. Since *L. casei* Shirota is a microorganism used in fermented milk production, it has been stated that its application in an aerosol or spray form can be safe and beneficial against respiratory tract infections.^66^

#### 2.3.7 Lung Cancer

Lung cancer is a type of cancer, with a high incidence among cancer types. Compared to all cancer deaths, approximately 1/4 is lung cancer-related mortality. It was determined that high rates of *Granulicatella*, *Abiotrophia*, *Streptococcus*, *Veillonella*, *Megasphaera*, and *Selenomonas* were found in BAL and phlegm samples of lung cancer patients. Altered bacterial diversity in the lung microbiome has been associated with different stages of cancer.^39^

*L. rhamnosus GG* was given by nebulization to mice with cancer models. It has been observed that the applied probiotics reach the lungs and reduce lung metastasis. Furthermore, *Bifidobacterium* administration after antibiotic treatment also helped reduce cancer cells. In addition, administration of aerosolized *Lactobacillus* caused tumor reduction, stimulation of T cells and NK cells, and thus the immune system’s response. As a result, inhaled probiotics, in addition to the current treatment, may aid in increasing the effectiveness of chemotherapy and eradicating cancer.^67^

#### 2.3.8 COVID-19

Studies conducted with COVID-19 patients show that the gut microbiota has changed significantly and that opportunistic pathogens have increased dramatically, despite the decrease in healthful bacteria. Significant reductions in *Lactobacillus* and *Bifidobacterium* species, the primary source of probiotics, were detected in the gut of COVID-19 patients. It is known that the gut microbiota can affect the response of the host to respiratory viral infections. A healthy gut microbiota increases the number of CD-8 T cells and the antiviral response of the lungs. Therefore, it is anticipated that the gut microbiota may influence the symptoms and severity of COVID-19.^68,69^ Apart from this, it was observed that the serum vitamin D ratio increased when *Lactobacillus* was administered orally. The increase in the vitamin D ratio is explained by lowering the pH of the intestinal epithelium thanks to lactic acids and increasing vitamin absorption. It is stated that vitamin D can provide a milder disease case of COVID-19 infection due to its immunomodulatory effects and inhibit cytokine storm by simultaneously supporting innate immunity and avoiding the excess of adaptive immunity.

**Figure 4:**
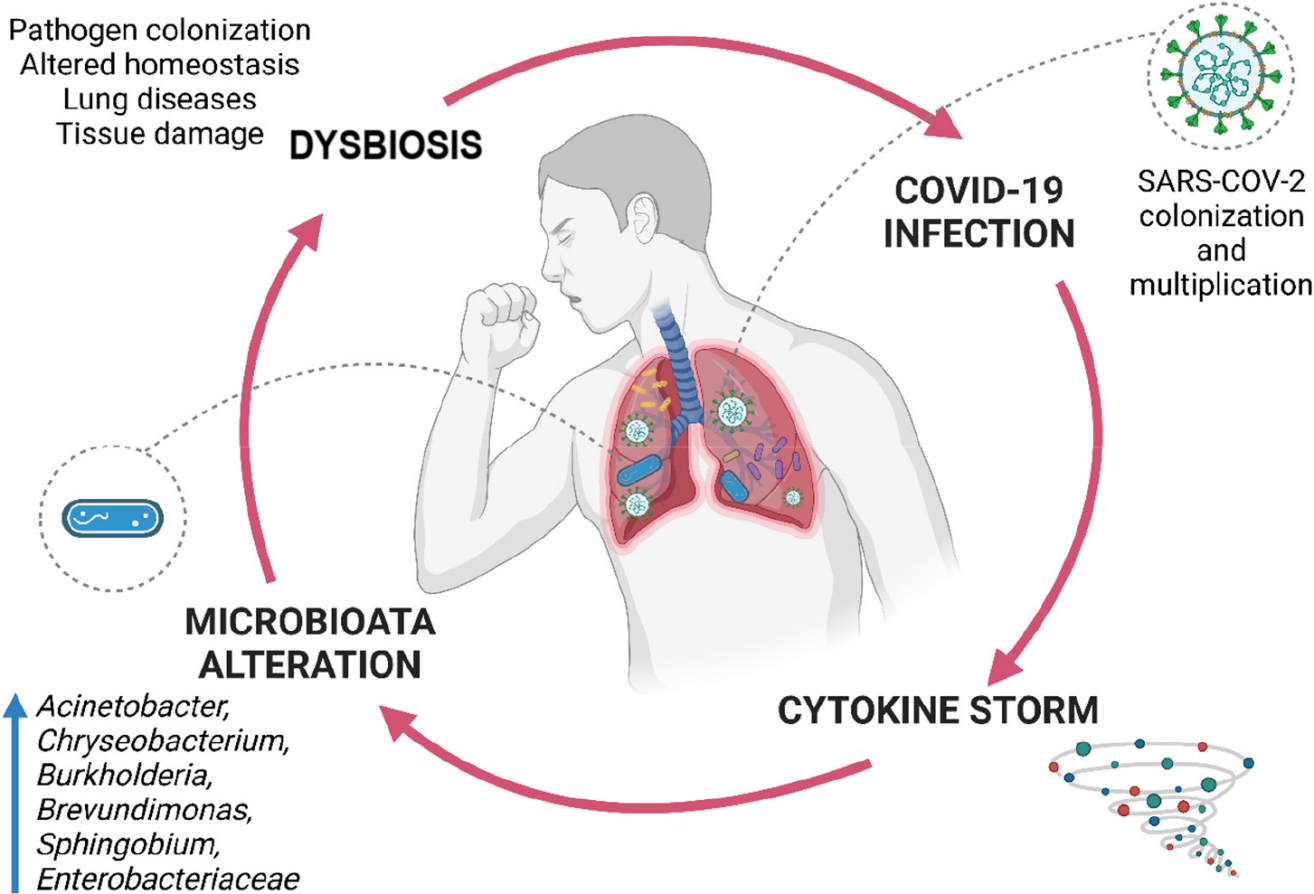
Relationship between lung microbiota and COVID-19 infection. The presence of dysbiosis in the lung can make a patient vulnerable to COVID-19 disease. As a result of the COVID-19 infection, cytokine storms and changes in lung microbiota can be seen. Microbiota shifts may also cause dysbiosis.

Probiotics can also interact with ACE-2, the primary receptor of SARS-COV-2, releasing ACE-inhibitory peptides with a high affinity for this receptor. This interaction is essential for the course of the disease.^70^ In addition to the gut microbiota, it has been determined that the lung microbiota play a vital role in the course of the infection. The lung microbiome of COVID-19 patients has been found to be significantly different compared to that of healthy individuals. *Acinetobacter*, *Chryseobacterium*, *Burkholderia*, *Brevundimonas*, *Sphingobium*, and *Enterobacteriaceae* ratios were higher in the patient’s lungs than they should be (Figure 4). Few studies showing the association of probiotics with COVID-19 are based on previous coronavirus and viral infections. Preclinical studies have established beneficial effects of oral or nasal administration of probiotics such as prolonged lifespan, reduced weight loss, reduced viral load in the lungs, and minimal bronchial epithelial damage. Probiotics prevent the immune response from being more or less, ensuring a balanced immune response. When the immune response is less than expected, the body’s ability to fight against infection is limited. When the immune response is higher than expected, a “cytokine storm” is observed (Figure 4). Therefore, probiotic support may reduce the severity of COVID-19 morbidity and mortality.^70^ In addition, after taking samples from COVID-19 patients with the BAL method, the microorganism species can be determined by performing 16S rRNA sequencing, and the immune response can be increased by stimulating the local immunity by administering the missing microorganisms into the lung.^43^

## 3 Mechanism of Action of Probiotics

**Figure 5:**
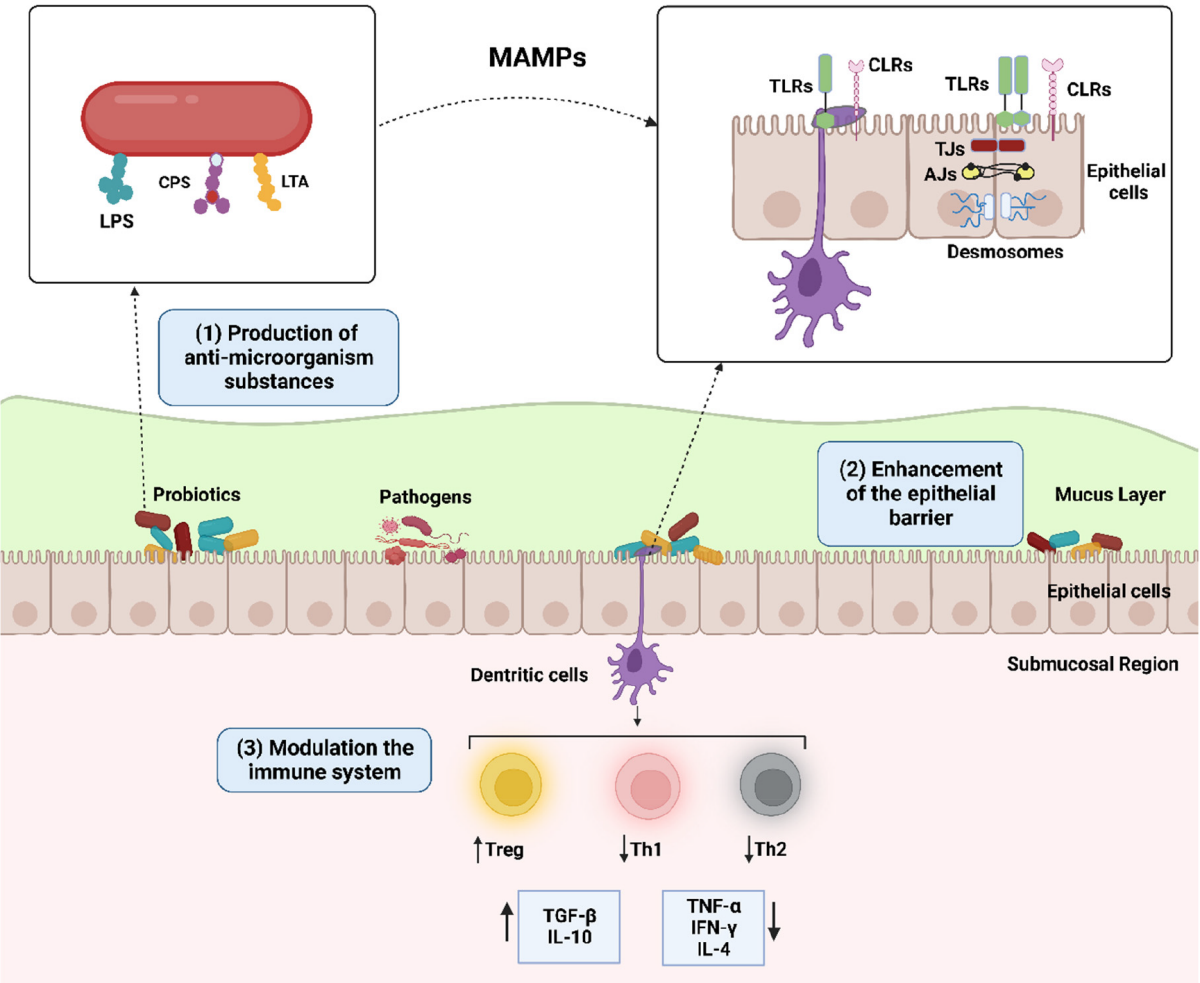
Primary mechanisms of action of probiotics. (1) Probiotics produce antimicrobial substances, (2) can enhance the barrier properties of epithelium enhancement of epithelial barrier by an interaction between MAMPs (i.e., LPS, CPS, and LTA) on the surfaces of probiotics and pattern recognition proteins on the epithelial barrier or modulation of intercellular junctions such as TJs, AJs, and desmosomes, and (3) can modulate the immune responses by interacting with dendritic cells. LPS: lipopolysaccharide; CPS: cell-wall–associated polysaccharide; LTA: lipoteichoic acid; MAMPs: microorganism-associated molecular patterns; TLRs: toll-like receptors, CLRs: C-type lectin receptors; TJs: tight junctions; AJs: adherence junctions. derived with permission from Martens et al.^73^ Copyright 2018 Wiley.

The protective effects of probiotics against pathogenic microorganisms are mainly based on three biological processes: (i) antimicrobial activity, (ii) supporting epithelial barrier properties, and (iii) immunomodulation (Figure 5). The antibacterial activity of probiotics is associated with the production of antimicrobial agents such as bacteriocins, which compete for a limited number of substrates and cellular adhesion sites, inhibit the production of virulence factors, hydrogen peroxide, and organic acids that acidify the ecosystem.^71,72^ Organic acids, especially acetic acid and lactic acid, have an inhibitory effect against Gram-negative bacteria. Acids that pass into the pathogen cell dissociate within the cell, lowering the intracellular pH. As a result, the ionized form of the organic acid accumulates in the cell and ultimately causes cell death. Bacteriocins are produced by Gram-positive bacteria (usually *Lactobacilli*) and are peptides active against bacteria. Bacteriocins show their antibacterial activity by destroying the target cell by inhibiting the pathogen cell wall synthesis or forming pores.^72^ Studies have also demonstrated the antiviral effect of probiotics against various intestinal, respiratory, and urogenital diseases caused by viruses. Direct interaction with virus cells and inhibition of replication, blocking of receptor sites in host cells, stimulation of immunity, and production of antiviral metabolites are suggested to explain the antiviral activities of probiotics.^69^

Intercellular junctions, tight junctions (TJs), adherence junctions (AJs), and desmosomes in the epithelium form a physical barrier and ensure the integrity of the epithelium. Probiotics regulate epithelial barrier function by increasing the expression levels of genes associated with these sites between epithelial cells.^73^ In addition, stimulating mucus secretion and releasing antimicrobial peptides such as β-defensins by probiotics also strengthen the barrier function. Defensins form pores in the bacterial membrane, disrupting the integrity of the membrane and accelerating bacterial lysis.^72^

Airway epithelial cells are equipped with pattern recognition receptors (PRRs), such as Toll-like receptors (TLRs), C-type lectin receptors (CLRs) that rapidly sensitize to microbial threats and initiate an immune response, and cytokine receptors, including TNFR1, which allow them to respond to signals generated by immune cells, such as airway macrophages. TLRs are expressed on immune and nonimmune cells, such as B-cells, NK cells, dendritic cells (DCs), macrophages, fibroblast cells, epithelial cells, and endothelial cells. PRRs recognize microorganism-associated molecular patterns (MAMPs) present on the probiotic cell surface, such as lipoteichoic acid (LTA), lipopolysaccharide (LPS), and cell wall-associated polysaccharide (CPS). Probiotics can modulate the local and systemic immune response in a species-specific manner via MAMPs. Probiotics interact with DCs located between epithelial cells or in the submucosal region. As a result of this interaction, regulatory T cells (Treg) are activated. Treg cells protect the epithelial barrier by producing TGF-β and IL-10. Probiotics balance between Th1 and Th2 responses resulting in the restoration of immune homeostasis (Figure 5).^72−74^

The following section discusses the technologies for preparing dosage forms of probiotics. These technologies will serve as the foundation for developing dry powder inhalers or intranasal colloidal solution or suspension formulations for targeting the respiratory tract and evaluating parameters when working with probiotics.

## 4 Probiotic Technology

Probiotics have antimutagenic, anticarcinogenic, antidiarrheal, and antimicrobial effects. Additionally, they have health benefits, such as modulation of the immune system, improved lactose metabolism, lower serum cholesterol, improvement of IBD, GI infections, ulcerative colitis, Crohn’s disease, and suppression of *Helicobacter pylori* infection.^75−77^ The current administration of probiotics for these purposes includes (i) regular use at low doses for prophylaxis to maintain the continued availability and efficacy of the probiotic in the host microbiota and (ii) the use of relatively high doses for the treatment of microbiota dysbiosis at the site of infection or to boost interaction with immunologically responsible host tissues.^69^ Many probiotic products, particularly dairy products, are available today. In addition, a few probiotic preparations are licensed as drug products. Probiotic dosage forms include tablets, capsules, oral films, chewable tablets, and sachets for oral administration. There are also probiotic-containing products in the form of vaginal capsules and tablets. Moreover, probiotic-containing cosmetic brands are trendy. Formulation studies comprising probiotics are generally intended for oral administration; however, studies for different administration routes such as nasal, respiratory, rectal, dermal, and transdermal are also available.^26,38,78−83^

Since the effects of probiotics are strain-specific, it is essential to determine the genus and species of probiotic bacteria to benefit health. For the probiotic to be effective in the body, a probiotic product should have a minimum concentration of 10^6^–10^7^ CFU/g.^75^ The doses to be given daily are generally 5 × 10^6^–6 × 10^6^ CFU/day for children and 10 × 10^6^–20 × 10^6^ CFU/day for adults. Side effects of probiotics are rare, and no significant drug interactions are known.^84^ Nonetheless, the risk of bacteremia and endocarditis should be considered in immunosuppressed individuals, diabetic patients, and patients who have recently undergone surgery. Other risks include the transmission of antibiotic-resistance genes between commensal bacteria and probiotics, as well as harmful immunomodulation effects in pregnant women and newborns.^71^

**Figure 6:**
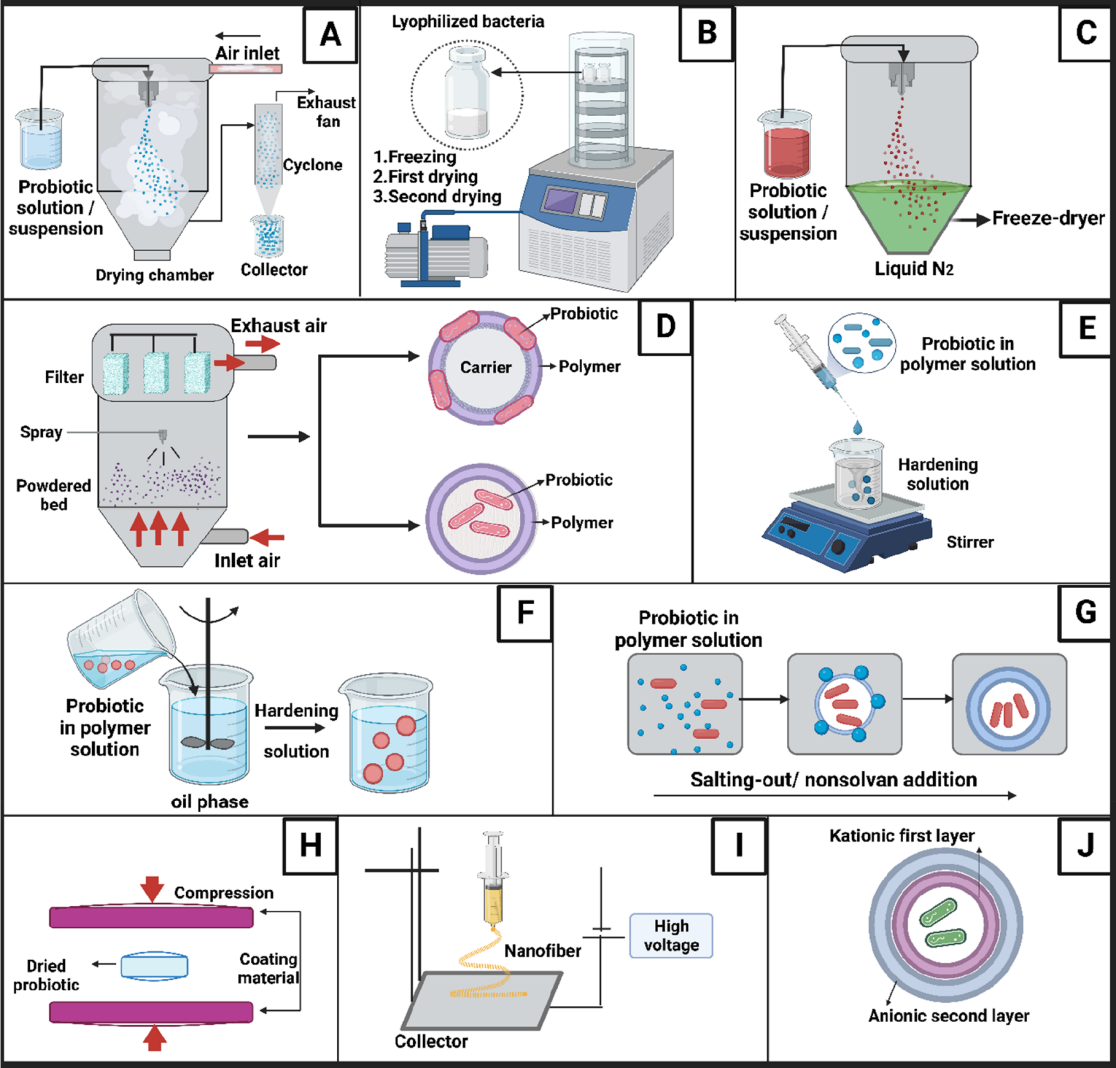
Production methods used for probiotic encapsulation. (A) Spray-drying, (B) lyophilization, (C) spray-freeze-drying, (D) fluidized bed drying, (E) extrusion, (F) emulsification, (G) coacervation, (H) coating by compressing, (I) electrospinning, and (J) layer-by-layer coating.

Assurance of the viability and stability of probiotics both during the production process and throughout their shelf life is crucial. The stability of probiotics is affected by temperature, pH, water content, oxygen, chemicals used, and environmental microorganisms.^85^ Factors such as bile acids in the body, the activity of digestive enzymes, high ionic strength, stomach movements, and fatty diet lead to a decrease in the viability of the bacteria.^83^ In this context, encapsulation of probiotics is preferred as a technology that can provide the necessary protection. Encapsulation protects probiotics from the host’s immune system and environmental factors, prevents probiotic viability loss during GI tract transit, and improves storage stability.^86−88^ Chitosan, alginate, starch, gelatin, kappa-carrageenan, lipids, whey protein, carnauba wax, gums such as xanthan, acacia, gellan, locust bean, and enteric polymers such as hydroxypropyl methylcellulose phthalate and cellulose acetate phthalate are used as carrier materials for the microencapsulation of probiotics.^89−92^ Probiotics can be added to formulations as live or heat-inactivated microorganisms. Different pharmaceutical methods, such as spray-drying, lyophilization, fluid bed drying, extrusion, emulsification, coacervation, electrospinning, coating by compression, adsorption, and layer-by-layer methods, are applied for the encapsulation of probiotics (Figure 6).^65,75,83,93,94^

### 4.1 Spray-Drying

In the spray-drying method, after mixing the aqueous/oily solution of probiotics and the solution of the encapsulation material, the material is sprayed into the drying chamber as droplets of controlled diameter. While the solvent/dispersion liquid evaporates through the drying air circulating at the set temperature in the drying chamber, the droplets turn into solid particles while preserving their original shape (Figure 6A).^95^ In this method, the concentration of the sprayed dispersion and the inlet air temperature are critical parameters.

In a study by Riveros et al., the *L. acidophilus* strain isolated from the human vagina was suspended in whey permeate or skim milk and spray-dried to improve probiotic properties and cell viability during storage. An increase in the drying temperature in the process decreases the bacterial viability. A product that provides the maximum viability of probiotics, together with long storage stability and less than 10% moisture content, was obtained at 60 °C and below outlet temperatures in a spray dryer. It has been found that adding guar gum to the formulation reduces the adhesion of the product to the dryer walls. The use of whey permeates provided 10^10^ CFU/g live microorganisms. A feed concentration of 12–30% was found to be the most appropriate in formulations. The probiotic bacteria remained viable for up to 2 months at 4 °C and stayed stable for 1 week at 25 °C.^96^

On the other hand, in a study by Avila-Reyes et al., *L. rhamnosus* was coated with a spray-drying process in the presence of rice starch and inulin as prebiotic materials. The recovery after drying was found to be 65–74% when rice starch was used and 43–54% when inulin was used. Furthermore, when different inlet temperatures and solid concentrations were tested, the most effective formulations were achieved with a low inlet temperature (135 °C) and high solid concentration (20%). As a result, both prebiotics were found suitable for using *L. rhamnosus* during spray-drying.^97^

*Bifidobacterium longum B6* and *Bifidobacterium infantis CCRC 14633* were encapsulated by spray-drying methods using gelatin, starch, skim milk, and guar gum. The reduction in their viability was investigated by exposing them to an acidic gastric medium and bile salts for 12 h. Encapsulation of *B. infantis CCRC 14633* cells using guar gum was found the most effective, with a 0.57% reduction in viability and a 2.24 log CFU/mL in colony reduction of probiotics.^98^

Jokicevic et al. prepared dry probiotic powder to make a nasal spray form by reconstitution. Centrifuged probiotic bacteria (*L. casei AMBR2*) were added to the polymer (hydroxypropyl methyl cellulose-HPMC or xanthan gum)–disaccharide/sugar alcohol solution and spray-dried together. Nasal spray formulations containing sucrose+xanthan gum, isomalt+xanthan gum, trehalose+HPMC, and lactose+HPMC provided a high survival ratio after 28 weeks (10^9^ CFU/g) at 4–8 °C and 7 days after reconstitution. Additionally, all these formulations showed high adhesion to Calu-3 cells and antimicrobial activity against URT pathogens.^99^

Another application area of the spray-drying process is obtaining the powder form of low-melting-point lipid materials by spray-chilling in the cold air stream, while in the hot melt state. Spray-chilling was applied to encapsulate probiotics in solid lipid microparticles. *L*. *acidophilus* probiotics were homogenized in melted, fully hydrogenated palm and palm kernel oil with or without prebiotics, inulin, and polydextrose. The prepared oily suspension was spray-chilled as solid lipid microparticles (SLMs, 1–4 μm). Polydextrose improved the potential of the symbiotic SLM in terms of the probiotic’s protection, release, and stability. When SLMs were immersed into enzyme-containing media, simulated gastric fluid (SGF) for 2 h and then simulated intestinal fluid (SIF) for 3 h, SLMs provided the delayed release of probiotics in simulated intestinal fluid (SIF) during fat digestion. SLMs containing polydextrose ensured the stability of the probiotic for 120 days at 7 °C with 11% humidity and −18 °C.^100^

### 4.2 Lyophilization

This technique involves freezing probiotics using a carrier material at low temperatures and then sublimating the solvent under a vacuum. In this method, osmotic pressure differences and mechanical stress induced by the formation of ice crystals may decrease probiotics’ viability during freezing and sublimation. The cryoprotectants, such as polyols and sugars, are added to the carrier materials to protect the probiotics (Figure 6B).^75,83^

**Figure 7:**
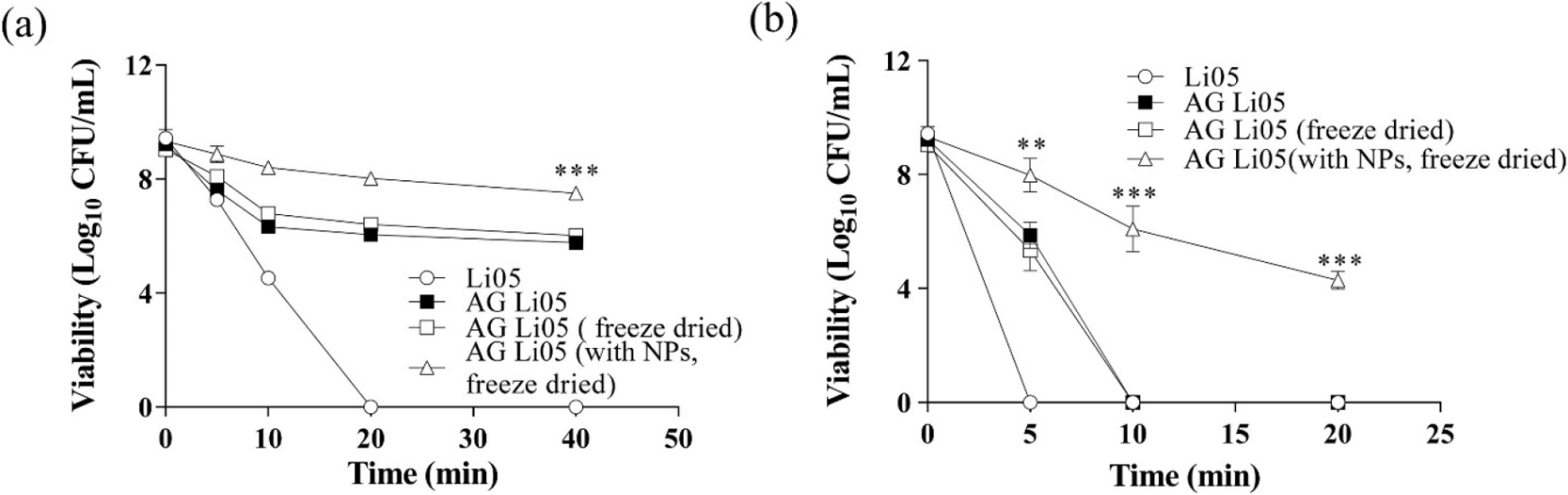
Probiotic viability was observed from freeze-dried AG Li05 with free probiotic Li05, AG Li05, freeze-dried AG Li05, and MgO NPs in SGF (a) and SIF (b). Reproduced with permission from Yao et al.^87^ Copyright 2018 Elsevier.

Yao et al. encapsulated the probiotic *Pediococcus pentosaceus Li05* in sodium alginate-gelatin (AG) microgels with MgO nanoparticles (NPs) using the lyophilization method.^87^ First, microorganisms were cultured, centrifuged, and suspended in a biopolymer gelling solution of alginate–gelatin and MgO NPs. Then, this suspension was sprayed into the CaCl_2_ solution by using an electrostatic microencapsulation unit to harden the droplets. Finally, samples of probiotic-loaded microgels were prefrozen in a–80 °C freezer overnight and then lyophilized in a freeze-dryer. It was observed that MgO NPs in the AG microgels adsorbed to the porous structure of the microgels, providing mechanical rigidity and a neutral pH, thus increasing the viability and stability of the probiotic. The decrease in probiotic viability in MgO NPs-loaded AG Li05 microgels after 40 min of incubation in SGF and SIF was less than 2 log_10_ CFU/mL (Figure 7).

### 4.3 Spray-Freeze-Drying

**Figure 8:**
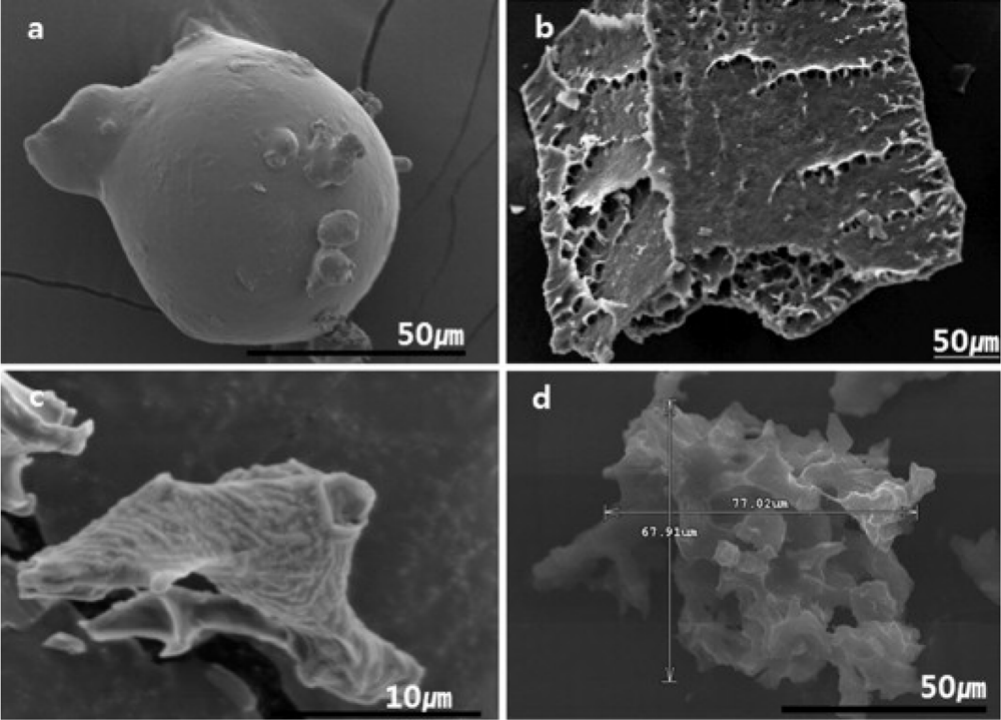
SEM image of *L. casei* powders prepared by (a) SFD with 1% glucose solution, (b) freeze-drying, (c) SFD with BPW solution without annealing, and (d) SFD with BPW solution with annealing. (Reproduced with permission from Her et al.^101^ Copyright 2015 Elsevier.

Spray freeze-drying (SFD) is a relatively new method that combines spray-drying and freeze-drying processes without heat application for manufacturing dry powder products (Figure 6C). Her et al. prepared the suspension of harvested probiotic *L.casei* cells in aqueous solutions containing different concentrations of glucose and sucrose as protectives and in buffered peptone water (BPW). This suspension was sprayed as small droplets under a controlled air pressure and spray rate into liquid nitrogen. The vessel containing liquid nitrogen with the probiotics was transferred to a deep freezer to anneal (at −15 °C for 3 h and then −40 °C for 1 h) and to evaporate the liquid nitrogen. Afterward, the sample was transferred to a freeze-dryer. The dry powder form of probiotics obtained by SFD had spherical shapes and smaller particle sizes than freeze-dried particles (Figure 8A,B). In addition, it was observed that annealing in SFD resulted in the formation of highly porous and spherical particles (Figure 8C,D). The probiotic viability in the powder obtained from the suspension containing 1% glucose with SFD was 99.1%.

Semyonov et al., on the other hand, achieved a high viability rate (>60%) by encapsulating *L. paracesei* using maltodextrin and trehalose in the SFD process. Trehalose was found to be an important agent in preserving cell viability during freezing and drying due to high osmotic pressure.^102^

### 4.4 Fluidized Bed Drying

One of the technologies used for drying probiotic-matrix mixtures is the fluidized bed method (Figure 6D). The coating material and probiotic dispersion are sprayed on the porous carrier substrates while fluidized with compressed air. Many loose and powdered materials have been used as substrates (carriers) such as wheat flour, skimmed milk powder, casein, maltodextrin, starch, microcrystalline cellulose, inulin, and NaCl. In addition, probiotic pellets that are freeze-dried or obtained by centrifugation can be coated with different polymers by the fluidized bed method.^103^

*L. acidophilus ATCC 4962* in 2% (w/v) skim milk was granulated by mixing with wet mass composed of corn starch, lactose monohydrate, microcrystalline cellulose, and, as a binder, povidone water solution (2%, w/w). After drying granules in an oven at 38 ± 2 °C for about 18 h, the granules were enteric coated with Eudragit L30D-55 polymer by a fluid bed dryer. The Eudragit L30D-55 coated formulation was developed to protect probiotics in the stomach while delivering them to the intestines. For the top-spray fluid bed drying process, the inlet air temperature, fluidized air flow rate, atomizer pressure, and spray rate were optimized as critical parameters that may affect the viability of probiotics during the coating process.^104^

In a study by Poddar et al., *L. paracasei 431* bacteria were dried using three different methods to evaluate the effect of drying techniques on the stability of probiotics: spray drying, lyophilization, and fluid bed drying. The suspension of *L. paracasei 431* in reconstituted milk powder in water was used for drying with the first two methods. For fluidized bed drying, freshly harvested probiotic pellets were mixed manually with whole milk for 10 min to form a uniform mass before drying. The viability of probiotics stored at 25 °C for 105 days was examined. Consequently, the fluidized bed system provided better protection for the probiotics. The reason for this is thought to be the low porosity of the particles, the formation of larger agglomerates, and the low water absorption feature. Lower water absorption causes the probiotic powder to remain stable for longer.^105^

### 4.5 Extrusion

Extrusion, one of the microencapsulation techniques, is used for the preparation of highly dense microcapsules. In the extrusion technique, an aqueous solution of hydrocolloids such as alginate and carrageenan is combined with the probiotic (lyophilized or slurry) to form an intense dispersion. This dispersion is taken into an extruder or injector and dropped into a hardener or gelling solution, such as CaCl_2_ (Figure 6E). The shape and size of the droplets depend on many factors, such as the tip of the dripped syringe, the distance to the dripped solution, and the device used.^95^ In a study in which *L. casei* was encapsulated step by step with sodium alginate, chitosan, and carboxymethyl chitosan by the extrusion method, the aggregation of microparticles was reduced, and the viability of microorganisms was preserved up to 10^7^ CFU/g.^106^

### 4.6 Emulsification

Preparing emulsions is another method for preserving probiotics in the biological environment and during storage (Figure 6F). In the study where sweet whey was used as an emulsifying agent in the secondary water phase, *L. rhamnosus* was encapsulated by a double emulsion technique (w_1_/o/w_2_). Double emulsions were incubated at pH 2.3 for 2 and 24 h in the presence of bile salts. It has been found that double emulsions maintain probiotic viability and even create a suitable environment for bacterial growth; the double emulsion droplet size and morphology did not change during incubation. The amount of sweet whey protein used in the formulation was a primary parameter in determining droplet size and preventing coalescence.^107^

The emulsification technique in forming beads provides faster and large scale production compared with the extrusion technique. In this technique, the alginate gelation is provided by adding its aqueous solution to an oil phase (liquid paraffin containing span 85) to form a water/oil (w/o) emulsion, followed by adding CaCl_2_; this procedure is called emulsification/external gelation. In emulsification and internal gelation, an insoluble calcium salt (i.e., CaCO_3_) is added to the inner water phase. After emulsion formation, an acid (i.e., glacial acetic acid) is added to the medium, resulting in the release of soluble Ca^2+^ for cross-linking of the alginate. Song et al. loaded a probiotic yeast cell (Y235) into alginate–calcium microcapsules coated with chitosan (ACMC). When the beads obtained by internal and external gelation techniques were compared, there was no significant difference in yeast cell viability (77% and 80%, respectively). Still, the emulsification/internal gelation technique was found to be more effective in terms of morphology, smaller particle size, and narrower size distribution than the emulsification/external gelation technique. In addition, it was observed that the cells proliferated, and cell leakage was less from ACMCs prepared by emulsification/internal gelation.^108^

### 4.7 Coaservation

In the coacervation technique, the polymer or polymers are precipitated by salting-out or the addition of nonsolvent (simple coacervation) or by phase separation due to the electrostatic interaction between two different charged polymers (complex coacervation) (Figure 6G). Oliveira et al. encapsulated *B. lactis* and *L. acidophilus* bacteria using a complex coacervation technique with pectin and casein as wall forming materials. The resistance of microencapsulated probiotic bacteria against the spray-drying process was evaluated, and their shelf life and in vitro acidity tolerance were examined. Microencapsulated bacteria were found to be more stable against the spray-drying process and SGF than free bacteria. In addition, the stability of microencapsulated *L. acidophilus* was better than *B. lactis* and *L. acidophilus*, and its viability was maintained for up to 120 days at 7 and 37 °C.^109^

### 4.8 Coating by Compression

The coating material is mantled on the core tablet after the dried probiotic powder had been compressed into a core tablet or pellet (Figure 6H). The pressure applied in this method can cause damage to the cell membrane and intracellular components of the probiotic bacteria. In a study, the powder of *L. acidophilus* obtained by freeze-drying in the presence of skimmed milk and sucrose was formed into 6 mm tablets under different pressures. When the applied pressure reached 90 MPa, the bacterial viability decreased to 85%. For this reason, probiotic tablets were prepared for compression coating at pressures up to 60 MPa. These tablets were compression coated using sodium alginate and hydroxypropyl cellulose as a coating material in a 10 mm die by direct compression at the pressure of 60 MPa. It was observed that the loss of probiotic viability was insignificant in this second compression due to the formulation and the applied processing conditions. Compression-coated tablets increased the stability of probiotics approximately ten times when stored at 25 °C for 30 days compared to plain probiotic powder or pellets.^93^

### 4.9 Electrospinning

Electrospinning produces nanofibers from polymer solutions in a high electric field created by a high voltage (Figure 6I). Poly(vinyl alcohol), poly(ethylene oxide), polyvinyl pyrrolidone, and chitosan as soluble polymers in water or mild acid (i.e., acetic acid) are frequently used to form probiotic-containing nanofibers by electrospinning. Due to rapid water evaporation, osmotic environmental change and applied voltage in the production of nanofibers may adversely affect the probiotic viability. Therefore, excipients such as prebiotics and cryoprotectants can be added to electrospinning solutions.^110^

By electrospinning, Yilmaz et al. obtained nanofibers containing *L. paracasei KS-199* and poly(vinyl alcohol)-sodium alginate (PVA/SA). The cells remained viable during the exposure to high voltage levels (22 kV) applied to the electrospinning process and storage in a refrigerator as nanofibers, indicating that the electrospinning process did not significantly affect the stability and metabolism of *L. paracasei*. The viability rates after incubation of nanofibers and nonencapsulated bacteria in SGF were 70.8% and 64.1%, respectively. The study results showed that encapsulation of *L. paracasei* in nanofibers by electrospinning technique has a protective effect on the cell structure.^111^

Another study aimed to prepare a nanofiber web with PVA and poly(ethylene oxide) (PEO) mixtures as a fast-dissolving dosage form of *L. Paracasei* in the oral cavity to protect dental health in periodontal diseases and to sustain a healthy microbiota in the mouth.^112^ The nanofibers were obtained by high-speed electrospinning from PVA and poly(ethylene oxide) (PEO) mixtures containing stabilizing excipients. Probiotic-loaded, smooth nanofibers with a diameter of about 1 μm were formed from polymer mixtures containing sugars such as glucose, sucrose, mannitol, trehalose, prebiotic inulin, or skimmed milk as stabilizing agents. It was observed that the number of cells decreased in the PVA–PEO fibers without additives, while a bacterial survival of over 80% was observed in PVA–PEO fibers containing trehalose, sucrose, and skim milk. This effect is explained by the fact that these substances reduce osmotic stress and their polar groups replace water molecules and protect the integrity of bacterial membranes and proteins. Generally, high probiotic viability was achieved for one year in nanofibers containing skim milk and mannitol stored at temperatures of 7 °C and below.

### 4.10 Layer-by-Layer Method

The layer-by-layer (LbL) method is an electrostatic coating process based on the ionic interaction between cationic and anionic polymers (Figure 6J). This coating is intended for the protection of probiotics from the acid environment and bile salts in the GI system and to ensure their proliferation/colonization in the intestine.^88^

*Bacillus coagulans* (BC), used for treating irritable bowel syndrome and colitis, was encapsulated by a layer-by-layer method using chitosan and alginate in two repeats (LbL-BC). The bacterial growth and proliferation ability continued as long as the coating integrity was maintained in LbL-BC. With the increase in the number of alginate–chitosan LbL coatings on the probiotic surface, the resistance of the probiotic to SGF and bile salts also increased. When the effect of layers on the mucoadhesion and growth of BC in isolated porcine small intestines and intestine-mimicking tissues from humans was examined, higher probiotic mucoadhesion from LbL-BC was observed than plain BC. This method is promising for introducing specific probiotics into the GI tract.^94^

*Saccharomyces boulardii* has been encapsulated with this technology using oppositely charged polymers, chitosan, and dextran sulfate. The viability of coated probiotics in SGF with enzyme was 7.19 ± 2.00 log CFU/100 mg, while the viability of uncoated probiotics was as low as 4.24 ± 1.41 log CFU/100 mg. The electrostatic interaction between polymers has been thought to help the yeast cell maintain its stability. In addition, multilayer encapsulation of microorganisms increases the effectiveness of the probiotic by making it more stable in the GI environment.^113^

In general, microencapsulation is the primary technology used to preserve the viability of probiotics and prepare their stable products. Fluidized bed and spray drying methods are frequently utilized as encapsulation techniques. Via the microencapsulation process, the probiotic bacteria are coated with a membrane or kept in the membrane so that they are transported to the site of action and released in a controlled manner from the encapsulating membrane, preserving the probiotics’ viability in the physiological environment. For this reason, the selection of the material used in the coating of the probiotics is essential. Prebiotics such as chitosan, inulin, pectin, starch, guar gum, xanthan gum, polydextrose, and sodium alginate are widely used. Prebiotics show a symbiotic relationship by selectively stimulating probiotic growth or activity and increasing probiotics’ effectiveness. Polymeric materials, for example, natural polymers such as casein, gelatin, and synthetic or semisynthetic polymers such as PVA, HPMC, MCC, and polymethacrylate-based (Eudragit) copolymers can provide the controlled release of probiotics.

## 5 Probiotic Targeting to the Lung and Future Perspectives

Lung physiology presents unique properties for drug targeting. It has features like high permeability, noninvasive application, large surface area for absorption, limited proteolytic activity, suitability for local and systemic treatment, reducing dose-related toxicity, and averting the first pass effect. Nonetheless, drug delivery systems applied to the lungs have drawbacks such as difficulty adjusting pH-isotonicity and aerodynamic particle size, sensitive physiological tolerability, and demanding manufacturing-license requirements. The pulmonary bioavailability of drugs is affected by the size and geometry of the drug delivery system, airway anatomy, and respiratory parameters. The ideal diameter for accumulation in the lung is 1–5 μm. Large particles are cleared by mucociliary clearance, whereas small ones are exhaled during breathing.^114,115^ Therefore, particulate drug delivery systems such as microcapsules, microspheres, liposomes, and niosomes are favorable to provide the required particle size.

Nebulizers can be used to deliver probiotics to the lungs from liquid formulations. Different nebulizer types exist as ultrasonic, jet, breath-actuated, and vibrating mesh. Among these, vibrating mesh nebulizers are advantageous as they are portable, easy to use, and provide effective particle diameter distribution.^116^ A soft mist inhaler is another device in which the liquid dosage form is aerosolized using mechanical force. A microchannel nozzle system sprays the aerosol mist, resulting in optimum droplet size between 1 and 5 μm. In addition, these inhalers are designed to achieve a high lung accumulation of around 50% and permit using pocket-size devices that patients could easily carry outside hospital or house settings.^117^ One limitation is that the drug should be soluble in water or water+ethanol not to obstruct the filters.^118^ Pressurized metered dose inhalers have restrictions on newly developed inhaler products due to the pressure on the use of propellants following the Montreal Protocol. Therefore, their production and usage should be carefully evaluated because of safety concerns. Applying dried powder probiotics by dry powder inhaler technology is another approach to target the lungs. The limitation of the formulation is the carrier requirement. Lactose is used as a carrier in approximately 90% of the dry powder inhalers on the market. The drug’s and lactose particle size ratio, humidity, electrostatic forces, and the surface properties of the drugs and lactose are among the critical parameters in formulation development. Although this technique is convenient, regarding stability, it is more gradual and laborious.

As mentioned before, probiotics raise the level of cells that have a role in immunity, such as NK cells, T cells, and antigen-presenting cells (APC), and the level of type-1 interferons and specific antibodies in the lungs. They are involved in the regulation of the dynamic balance of proinflammatory cytokines. Therefore, probiotics are essential in maintaining balance and preventing cytokine storm.^119^ Studies of targeting probiotics to the lungs demonstrate the effectiveness of probiotics in lung diseases. Intranasal or inhaler probiotic administration can be the preferred treatment option against antibiotic resistance.^59,120^ The COVID-19 pandemic has led researchers to conduct therapeutic and prophylactic studies directed toward the lungs. Considering the increasing studies on COVID-19 disease, it is thought that using probiotics in high-risk patients and healthcare workers may limit COVID-19 infection.^70^ Besides, microbiota transplantation from healthy lungs for people infected with COVID-19 could be considered.^43^ Despite all of this, there are some risks that unnecessary inhaled probiotic use may change the lung microbiota and cause diseases. Therefore, the critical issues related to probiotic inhalation are as follows;

i. More studies should be done on the changes in the microbiota due to the diseases, and a database should be established.
ii. Techniques for rapid microbiota sampling from patients should be promoted, and these techniques should offer high patient compliance.
iii. Analysis methods (innovative chip technologies, kits, etc.) should be developed to evaluate microbiota samples quickly (even in the home).
iv. Treatment with inhaled probiotics should be terminated as soon as the microbiota imbalance is resolved.

## 6 Conclusion

Probiotics have a tremendous role in maintaining lung health as well as gut health. It has been postulated that orally administered probiotics also have a protective effect against lung diseases. In addition, studies on probiotics administered via nasal and intratracheal routes have shown a modulation effect on lung diseases. The investigations have demonstrated the relationship between lung microbiota and lung infections. Despite all of the beneficial effects, studies on the delivery of probiotics by the pulmonary route are very few. Thus, future directions will include the development of formulations for effectively delivering probiotics to the lungs.

## Abbreviations

Ac-PGP: *N*-acetyl-proline-glycine-proline
ACE-2: angiotensin converting enzyme
ACMC: alginate-calcium microcapsules coated with chitosan
AJs: adherence junctions
APC: antigen-presenting cells
BAL: bronchoalveolar lavage
BC: *Bacillus coagulans*
BPD: bronchopulmonary dysplasia
BPW: buffered peptone water
CF: cystic fibrosis
CFU: colony forming unit
CLRs: C-type lectin receptors
COPD: chronic obstructive pulmonary disease
COVID-19: coronavirus disease 2019
CPS: cell-wall-associated polysaccharide
DCs: dendritic cells
FDA: Food and Drug Administration
GI: gastrointestinal
GRAS: generally recognized as safe
IBD: inflammatory bowel disease
IFN: interferon
IgE: immunoglobulin E
IL: interleukin
LbL: layer-by-layer
LDH: lactate dehydrogenase
LPS: lipopolysaccharide
LTA: lipoteichoic acid
MAMPs: microorganism-associated molecular patterns
MCP-1: monocyte chemoattractant protein-1
MPa: mega pascal
NK: natural killer
NPs: nanoparticles
LRT: lower respiratory system
Q-PCR: quantitative-PCR
PBS: phosphate-buffered saline
PEO: poly(ethylene oxide)
PRRs: pattern recognition receptors
PVA: poly(vinyl alcohol)
SA: sodium alginate
SARS-COV-2: severe acute respiratory syndrome coronavirus 2
SCFAs: short-chain fatty acids
SEM: scanning electron microscope
SFD: spray-freeze-drying
SGF: simulated gastric fluid
SIF: simulated intestinal fluid
SLMs: solid lipid microparticles
RSV: respiratory syncytial virus
Th1: T helper type-1
Th2: T helper type-2
TGF: transforming growth factor
TJs: tight junctions
TLRs: toll-like receptors
TNF: tumor necrosis factor
Treg: regulatory T cells
UK: United Kingdom
URT: upper respiratory system
US: United States
